# Midwives Registration Association

**Published:** 1894-01-27

**Authors:** Frederick H. Alderson

**Affiliations:** President of the General Practitioners' Association


### EDITOR'S LETTER-BOX.

[Our correspondents are reminded that proxility is a great bar to publication, and that brevity of style and conciseness of statement greatly facilitate early insertion.]

### MIDWIVES' REGISTRATION ASSOCIATION.

SIR,—I read with considerable interest your Annotation of this week's HOSPITAL on the debateable question, "Should Midwives be Registered?" to which my attention has been directed by one of our leaders in gynecology. The topic is one of special interest to me, holding office as one of the Executive Committee of the new society established for the promotion of registration, education, and supervision of midwives, and being also the President for the year of that rapidly-increasing society (both as to numbers and the importance of its work) the Medical Practitioners' Association —a society that most actively and successfully opposed the passing of the badly-conceived and unworkable Bill of 1892—and whose members many of them were conscientiously opposed to any legislation for the controlling of the practice of the time-honoured and, in her generation, useful midwife.

The question, therefore, that you ask your readers is of considerable importance, requiring—indeed, demanding—the thoughtful attention of the general practitioner.

In the attempt to solve the question it is well to remember the considerable time that our Government have already given to the Midwives Bill, and that two Select Committees have been appointed by the House of Commons. The report of the last one gives convincing evidence that there is urgent need in the public interest, and for the saving both of maternal and infant life, and for the prevention of considerable suffering and of many prolonged illnesses, caused or increased by the ignorance of a very large class of midwives, that there should be some useful and practical legislation that shall prevent this deplorable evil for the future, and preserve the health and morals of the public. I think, then, it may be accepted that there will be some Act of Parliament passed, and probably at no distant date, to remedy the evils that our Government have taken cognizance and deplore. Shall the profession, then, hold aloof, or, because we disapproved of a bad Bill, prevent the passing of a useful measure?

I read with pleasure that it is the opinion of the Editor of THE HOSPITAL that medical men will not be injured but benefited by the registration of midwives, and of this I feel sure, sir; unless by the apathy and indifference of the profession they leave the matter entirely in non-medical hands, our legislators are only too willing to give us voice and to hear and be advised by the opinion of the profession. As a member of the Executive Committee of the Midwives' Registration Association, I believe I am acting in the interest of the general practitioner, as well as of the public, and at one of our last meetings I was instrumental in carrying unanimously a proposition that the general practitioner should be eligible for the office of examiner to the Midwifery Board. Allow me also to direct the attention of your many readers to the fact that it is only proposed to register duly qualified, educated, and suitable women as midwives; and these are to be under the control and supervision of medical men. Vested rights are cared for, and would rightly be insisted upon by Parliament; but at the same time only those midwives of good character and in *bonâ fide* practice at the passing of the Act would be allowed to continue in practice of their calling, and these women would be placed on a separate list and would forfeit even this privilege if they used the distinction of registration or implied the new register.

We advocate that registration should be reserved for the qualified and competent midwife, the other or inferior class of present untaught midwives would quickly become smaller and smaller, and finally cease to exist; and, remember, whether any Act is passed or not these midwives will exist, but in the latter case for the greater detriment of the public.—Yours very obediently,

FREDERICK H. ALDERSON, M.D.,
President of the General Practitioners' Association.

